# Prenatal prediction of neonatal respiratory morbidity: a radiomics method based on imbalanced few-shot fetal lung ultrasound images

**DOI:** 10.1186/s12880-021-00731-z

**Published:** 2022-01-04

**Authors:** Jing Jiao, Yanran Du, Xiaokang Li, Yi Guo, Yunyun Ren, Yuanyuan Wang

**Affiliations:** 1 Department of Electronic Engineering, Fudan University, No. 220, Handan Road, Yangpu District, Shanghai, 200433 China; 2 Key Laboratory of Medical Imaging Computing and Computer Assisted Intervention of Shanghai, Shanghai, 200433 China; 3 Department of Ultrasound, Ruijin Hospital, Shanghai Jiaotong University School of Medicine, No.197 Rui Jin 2nd Road, Shanghai, 200025 China; 4 Department of Ultrasound, Obstetrics and Gynecology Hospital of Fudan University, No. 128, Shenyang Road, Shanghai, 200090 China

**Keywords:** Neonatal respiratory distress syndrome, Transient tachypnea, Prenatal ultrasonic diagnosis, Fetal lung ultrasound image, Class imbalance, Ensemble learning

## Abstract

**Background:**

To develop a non-invasive method for the prenatal prediction of neonatal respiratory morbidity (NRM) by a novel radiomics method based on imbalanced few-shot fetal lung ultrasound images.

**Methods:**

A total of 210 fetal lung ultrasound images were enrolled in this study, including 159 normal newborns and 51 NRM newborns. Fetal lungs were delineated as the region of interest (ROI), where radiomics features were designed and extracted. Integrating radiomics features selected and two clinical features, including gestational age and gestational diabetes mellitus, the prediction model was developed and evaluated. The modelling methods used were data augmentation, cost-sensitive learning, and ensemble learning. Furthermore, two methods, which embed data balancing into ensemble learning, were employed to address the problems of imbalance and few-shot simultaneously.

**Results:**

Our model achieved sensitivity values of 0.82, specificity values of 0.84, balanced accuracy values of 0.83 and area under the curve values of 0.87 in the test set. The radiomics features extracted from the ROIs at different locations within the lung region achieved similar classification performance outcomes.

**Conclusion:**

The feature set we designed can efficiently and robustly describe fetal lungs for NRM prediction. RUSBoost shows excellent performance compared to state-of-the-art classifiers on the imbalanced few-shot dataset. The diagnostic efficacy of the model we developed is similar to that of several previous reports of amniocentesis and can serve as a non-invasive, precise evaluation tool for NRM prediction.

## Background

Neonatal respiratory morbidity (NRM), mainly including respiratory distress syndrome (RDS) and transient tachypnea of the newborn (TTN), is a leading cause of morbidity and mortality in the preterm and early term [1]. The morbidity of NRM is correlated with fetal lung maturity [2]. Newborns with NRM are born with respiratory distress and even apnoea, which may lead to multiple complications, or even death. Glucocorticoids are used to treat fetuses at high risk of NRM to promote fetal lung maturation and can significantly reduce morbidity and mortality. However, recent studies have shown that glucocorticoid treatment has some side effects, such as short-term fetal heart rate variability (HRV) and fetal movements [3]. An accurate prenatal prediction of NRM is essential to avoid the overuse of glucocorticoids in normal fetuses.

Amniocentesis is an effective method for the prenatal prediction of NRM by assessing fetal lung maturity [4]. However, it is an invasive detection method with complicated and time-consuming operations and no uniform threshold for the prediction. Currently, amniocentesis is rarely used to make prenatal predictions. Instead, gestational age (GA) is usually assessed to make the prediction. Fetuses assessed to be born at 28–36.6 weeks are regarded as having a high risk of NRM because of fetal lung immaturity and will be treated with glucocorticoids. There is a high rate of false positives in view of NRM morbidity, which will cause side effects in newborns. In this context, it is particularly important to develop an accurate and non-invasive method for the prenatal prediction of NRM.

Ultrasound is a non-radiation and non-invasive technology that is widely used in prenatal diagnosis. The use of fetal lung ultrasound images to predict NRM as alternative to amniocentesis has been considered a useful method in recent studies [5]. In a recent study, quantitative texture analysis of fetal lungs (quantusFLM) was used to predict NRM [6]. The study was based on the European population and no related study for Asian populations. Moreover, the feature set used in their study only includes textural features and GA. There is suggestive evidence that gestational diabetes mellitus (GDM) in pregnant women may have adverse effects on lung development [7, 8]. On the other hand, due to low morbidity, NRM newborns, especially preterm and early-term newborns, are hard to obtain. The dataset for the study is usually imbalanced and few-shot. This phenomenon was not mentioned in their study. It is worth noting that imbalanced and few-shot datasets are common in clinical practice and will bring overfitting and bias, resulting in poor generalization for the classification model.

The purpose of this study was to develop a non-invasive method for the prenatal prediction of NRM based on the radiomics method with an imbalanced few-shot fetal lung ultrasound image dataset collected from Asian population. Fetal lungs were delineated as the region of interest (ROI), and radiomics features were designed and extracted from the ROI. Feature selection was performed to select representative radiomics features and combining with GA and GDM for modelling. The modelling method of data augmentation, cost-sensitive learning, ensemble learning, Random Under-Sampling with AdaBoost (RUSBoost) [9] and Synthetic Minority Oversampling Technique (SMOTE) with AdaBoost (SMOTEBoost) [10] were used to address the problems of imbalance and few-shot. Finally, the diagnostic efficacy of the model we developed was found to be similar to that of previous reports of amniocentesis.

## Methods

### Workflow

**Fig. 1:**
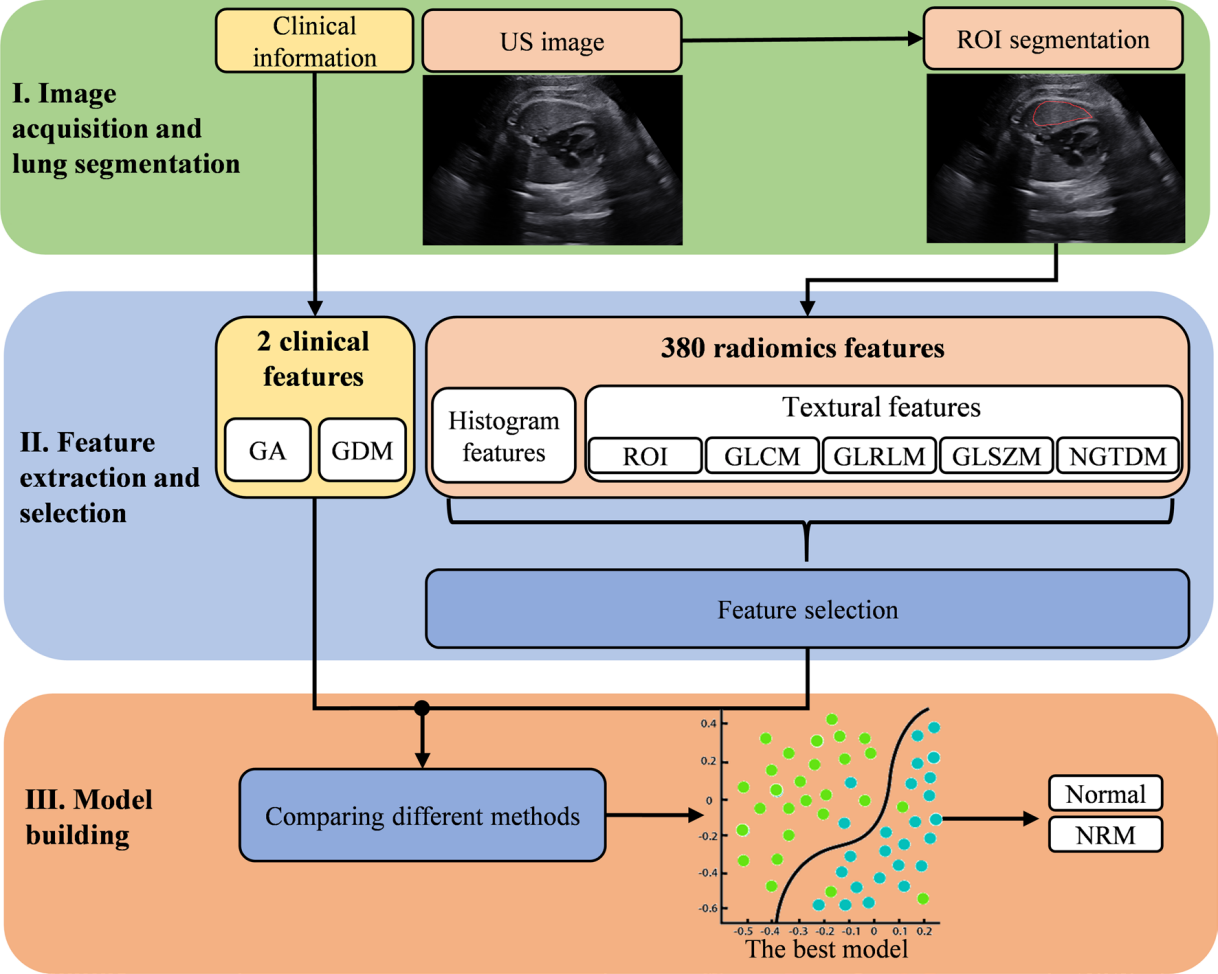
The Workflow of the entire study. Stage I: For each acquired fetal lung ultrasound image, the ROI inside the fetal lung is delineated by one physician and confirmed by another physician. Stage II: 308 radiomics features are extracted in the ROI of each image. Feature selection is performed on these radiomics features to select the most useful features. Stage III: the selected radiomics features are combined with the clinical features as the input to the classifier. With building and comparing classification models with different methods, the best model is finally selected to predict the risk value of NRM

The workflow for the entire study is summarized in Fig. 1. It can be divided into three parts: image acquisition and lung segmentation, feature extraction and selection, model building. First, for each acquired fetal lung ultrasound image, the ROI inside the fetal lung is delineated by one physician and confirmed by another physician. Then, 308 radiomics features are extracted in the ROI of each image. Feature selection is performed on these radiomics features to select the most valuable features. Finally, the selected radiomics features are combined with the clinical features as the input to the classifier. With building and comparing classification models with different methods, the best model is finally selected to predict NRM.

### Patients

**Fig. 2:**
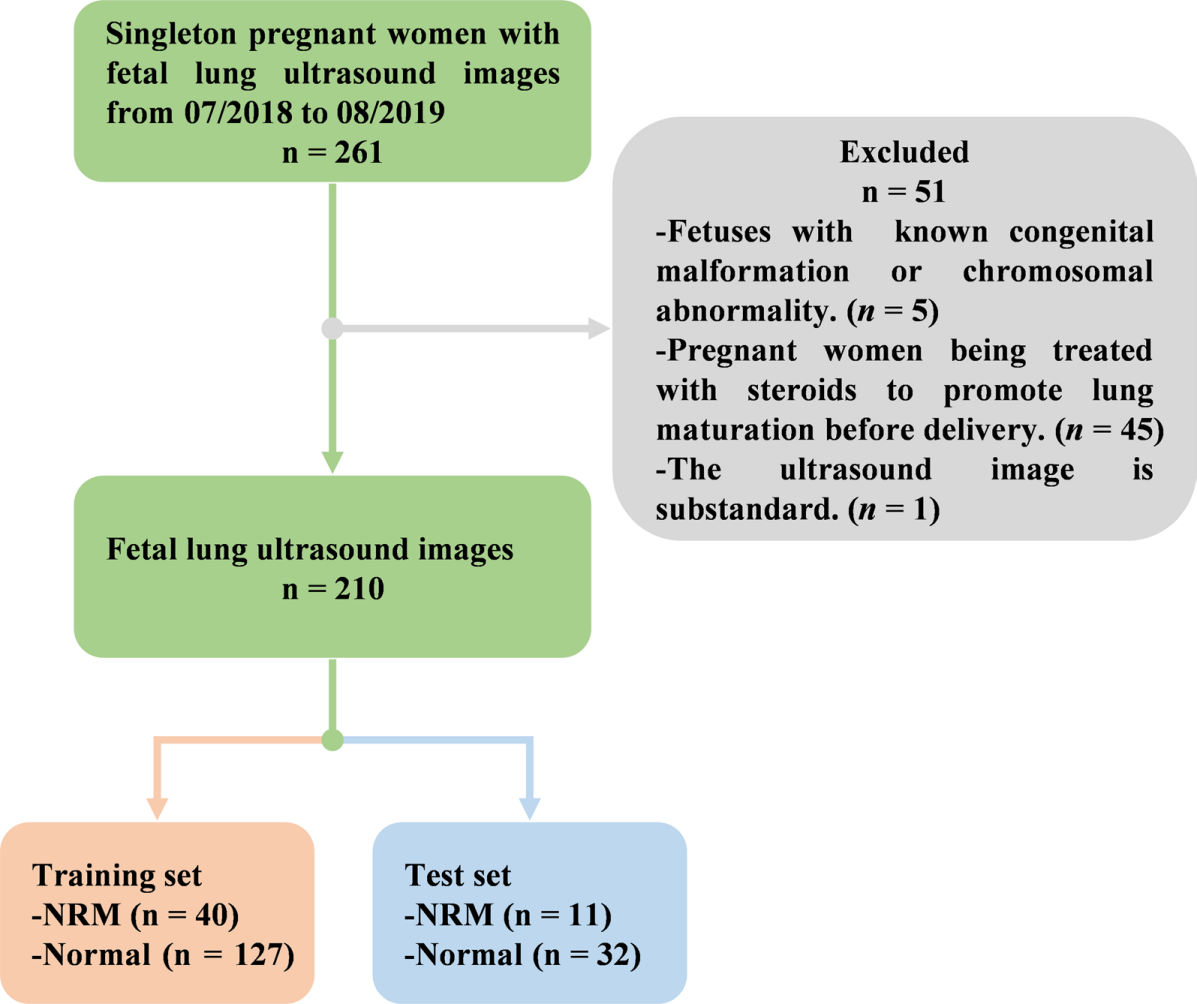
The flowchart of the selection process of the study population. Pregnant women who met the following criteria were enrolled in the study: (1) singleton pregnancy; (2) those with complete medical information who had undergone maternity examination and subsequent delivery in our hospital; (3) fetuses with no known congenital malformation or chromosomal abnormality; (4) those with no diabetes before pregnancy; and (5) those who had not been prescribed steroids before delivery. Finally, a total of 210 singleton pregnant women with 210 fetal lung ultrasound images were enrolled in our study and randomly divided into the training set and test set at a ratio of approximately 8:2. It is worth noting that we kept the same proportion of NRM and normal in both sets. The training set contains 167 images, of which 40 are NRM and 127 are normal. The test set contains 43 images, of which 11 are NRM and 32 are normal

From July 2018 to August 2019, a total of 261 fetal lung ultrasound images from 261 singleton pregnant women with GAs ranging from 28.0 to 38.6 weeks were collected from Obstetrics and Gynecology Hospital Affiliated to Fudan University, Shanghai, China. The flowchart for the study population is shown in Fig. 2. Pregnant women who met the following criteria were enrolled in the study: (1) singleton pregnancy; (2) those with complete medical information who had undergone maternity examination and subsequent delivery in our hospital; (3) fetuses with no known congenital malformation or chromosomal abnormality; (4) those with no diabetes before pregnancy; and (5) those who had not been prescribed steroids before delivery. Finally, a total of 210 singleton pregnant women with 210 fetal lung ultrasound images were enrolled in our study and randomly divided into the training set and test set at a ratio of approximately 8:2. It is worth noting that we kept the same proportion of NRM and normal in both sets. The training set contains 167 images, of which 40 are NRM and 127 are normal. The test set contains 43 images, of which 11 are NRM and 32 are normal.

This study was approved by the Ethics Committee of Obstetrics and Gynecology Hospital Affiliated to Fudan University, Shanghai, China. All data were collected and used with the consent of the pregnant women.

### Image acquisition and lung segmentation

All ultrasound images were obtained during routine prenatal ultrasound examinations within 72 h before delivery and performed by a radiologist with over 8 years of experience in obstetrics and gynaecology ultrasound imaging. The WS80A ultrasound system (Samsung, Korea) was used in this study for imaging. One scanner was used in this study: the Samsung CA1-7A curved array probe (frequency range 1.0–7.0 MHz, center frequency: 4.0 MHz).

Fetal lung ultrasound image acquisition was achieved using a transverse view of the fetal thorax at the level of the four-chamber view of the heart. The probe was adjusted to ensure that at least one of the lungs had no obvious acoustic shadowing from the fetal ribs. In order to obtain optimal image quality, the acquisition parameters, including depth, gain, frequency, time-gain compensation, and harmonics, were adjusted according to the relevant features of each pregnant woman and fetus. All the images were collected and stored in DICOM format (.dcm) for offline analysis.

**Fig. 3:**
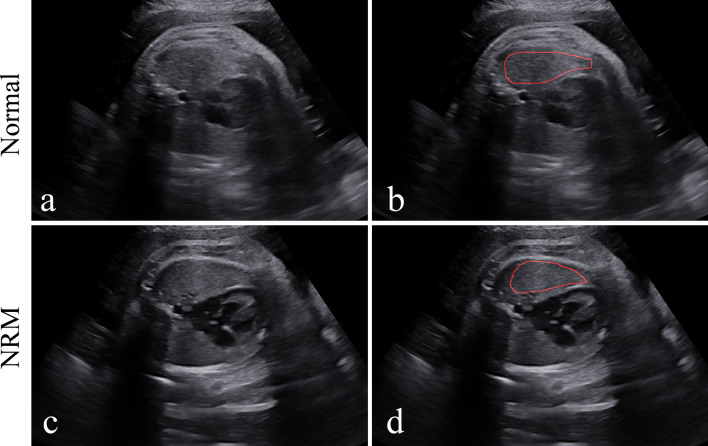
Examples of NRM and normal fetal lung ultrasound images and manual delineation. **a** An NRM fetal lung ultrasound image. **b** A normal fetal lung ultrasound image. **c** and **d** are the manual delineations of the ROIs

Figure 3 shows the manual delineation of the lung regions in the ultrasound images of a normal fetus and a fetus with NRM, respectively. All ROIs were selected in the homogeneous area inside the lung, with no vascular or rib shadows. It should be noted that the manual delineation of each fetal lung was delineated by one physician, which was reviewed and confirmed by another physician, both of whom were blinded to the medical histories of the pregnant women and neonatal outcomes.

### Feature extraction and selection

The feature design is the basis for building a practical and generalizable classification model. For ultrasound fetal lung images, the feature set should reflect subtle texture information in the ROI of the image and independent of the ROI's size and location to provide a robust description for clinical use. With the requirement for the feature set, a series of radiomics features were designed based on the image greyscale and texture, including 16 greyscale histogram features, 60 texture features, and 304 wavelet features.

Before feature extraction, the area inside the ROI where the feature extracted was min–max normalized into 0–255 to remove bias, scaling factors of the effect of different imaging parameters. To avoid the effect of outliers, we refer to Collewet's work [11] which calculates the maximum and minimum values after removing outliers for min–max normalization of images.

For the greyscale histogram features and texture features, we refer to the feature definitions as described by the Imaging Biomarker Standardization Initiative (IBSI) [12]. The bin-width is set to 1 to maintain detailed texture information. The 304 wavelet features were obtained by extracting 16 greyscale histogram features and 60 texture features separately on four components first-level decomposition (approximate, horizontal, vertical, and diagonal) of the original image's wavelet transform. We adopted the Daubechies wavelets 5 (db5) transform.

The extracted features have different value ranges, which will affect feature selection and modelling. In this study, we performed the min–max normalization on the extracted raw features to ensure effective selection and training in the following modelling process. Note that the maximum and minimum in the normalization are calculated from the training set and also used for normalization in validation and test sets. It is reasonable as the maximum and minimum of test samples are unseen in practice.

In addition, we used a priori clinical knowledge to improve the feature set's descriptive ability by adding two clinical features, GA and GDM, with are readily available and strongly correlated with NRM in relevant studies. The summary of the feature set is listed in Table 1, and the details of the features are as follows.

**Table 1:** The summary of the feature set we designed for predicting NRM

Feature type	Feature name	Feature number
Clinical information	(1) GA, (2) GDM	2
Greyscale histogram features	(3) Energy, (4) Entropy, (5) Kurtosis, (6) Mean, (7) Median absolute deviation, (8) Median, (9) Range, (10) Uniformity, (11) Variance, (12) Root mean square, (13) Skewness, (14) Deviation, (15) Histogram kurtosis, (16) Histogram mean, (17) Histogram variance, (18) Histogram skewness	16
ROI textural features	(19) Mean of contrast, (20) SD of contrast, (21) Mean of covariance, (22) SD of covariance, (23) Mean of non-similarity, (24) SD of non-similarity	6
GLCM textural features	(25) Energy, (26) Entropy, (27) Dissimilarity, (28) Contrast, (29) Inversed difference, (30) Correlation 1, (31) Correlation 2, (32) Homogeneity, (33) Autocorrelation, (34) Cluster shade, (35) Cluster prominence, (36) Maximum probability, (37) Sum of squares, (38) Sum average, (39) Sum variance, (40) Sum entropy, (41) Difference variance, (42) Difference entropy, (43) Information measures of correlation 1, (44) Information measures of correlation 2, (45) Maximal correlation coefficient, (46) Inverse difference normalized, (47) Inverse difference moment normalized	23
GLRLM textural features	(48) Short-run emphasis, (49) Long-run emphasis, (50) Grey-level non-uniformity, (51) Run length non-uniformity, (52) Run percentage, (53) Low grey-level run emphasis, (54) High grey-level run emphasis, (55) Short-run low grey-level emphasis, (56) Short-run high grey-level emphasis, (57) Long-run low grey-level emphasis, (58) Long-run high grey-level emphasis, (59) Grey-level variance, (60) Run-length variance	13
GLSZM textural features	(61) Small zone emphasis, (62) Large zone emphasis, (63) Grey-level non-uniformity, (64) Zone size non-uniformity, (65) Zone percentage, (66) Low grey-level zone emphasis, (67) High grey-level zone emphasis, (68) Small zone low grey-level emphasis, (69) Small zone high grey-level emphasis, (70) Large zone low grey-level emphasis, (71) Large zone high grey-level emphasis, (72) Grey-level variance, (73) Zone-size variance	13
NGTDM textural features	(74) Coarseness, (75) Contrast, (76) Busyness, (77) Complexity, (78) Strength	5
Wavelet features	(79–154) Approximation, (155–230) Horizontal, (231–306) Vertical, (307–382) Diagonal	304
Total feature number		382

(1) Clinical information: GA and GDM are strongly correlated with NRM [7, 8]. GA was determined by the last menstrual period and verified by first-trimester dating ultrasound (crown-rump length). According to the presence of GDM during pregnancy, these pregnant women were divided into Yes and No groups

(2) Greyscale histogram features: Describe the greyscale and histogram distribution of the ROI in fetal lung ultrasound images [13]

(3) Textural features: Describe detailed, invisible greyscale changes and associations in fetal lung ultrasound images

(a) ROI textural features: Describe the distribution of greyscale inside the ROI [14]

(b) Grey-level co-occurrence matrix (GLCM) textural features: Describe the specified spatial linear relationship between the frequencies of two greyscale intensities inside the ROI [15]

(c) Grey-level run-length matrix (GLRLM) textural features: Describe the roughness of the texture by calculating the run-length of the collinear image pixels of the same grey-level in a given direction inside the ROI [16, 17]

(d) Grey-level size zone matrix (GLSZM) textural features: Describe the uniformity of the small pixel population of the ROI [15, 18]

(e) Neighbourhood grey-tone difference matrix (NGTDM) textural features: Describe the difference between the greyscale of each image pixel and the greyscale of its neighbours inside the ROI [19]

(4) Wavelet features: Describe information that is not directly reflected by the greyscale and textural features of the original image. Every fetal lung ultrasound image was decomposed into four components: approximate, horizontal, vertical, and diagonal by wavelet transform (first-level decomposition). Then, the 76 features mentioned above were extracted separately on each component. Finally, a total of 304 wavelet features were extracted

Approximate, horizontal, vertical, and diagonal were decomposed from the image by wavelet transform (first-level decomposition)

*GA*: gestational age, *GDM*: gestational diabetes mellitus*, ROI*: region of interest (fetal lung region), *SD*: standard deviation, *GLCM*: grey-level co-occurrence matrix, *GLRLM*: grey-level run-length matrix, *GLSZM*: grey-level size zone matrix, *NGTDM*: neighbourhood grey-tone difference matrix

The feature selection method was used to select the most useful radiomics features as inputs of the classification model. We ranked feature importance to selected features by permuting out-of-bag data feature of random forest trees. If a feature is influential, permuting its values would influence the model error testing with out-of-bag data. The more important a feature is, the greater its influence will be [20].

### Model building

The class imbalance and small dataset will lead to overfitting and classification bias. In this study, we designed and evaluate performance of common methods on our imbalanced and few-shot dataset. The motivation of the comparison experiment is to compare the effectiveness of different modelling approaches on the imbalanced small dataset from both data and model perspectives.

To address the imbalance problem, we introduced a data balancing method, Adaptive Synthetic (ADASYN) [21]. ADASYN generates minority class pseudo-samples by linear interpolation to balance the dataset. Classifiers can then be trained on the balanced dataset without the effect of the class imbalance. This has been shown to be effective in some studies, but there is a lack of research on the small medical image datasets. We also introduced a classifier model cost-sensitive support vector machine (SVM) [22], which addresses the class imbalance problem by increasing the model's misclassification cost of the minority classes.

As for the problem of the low generalizability of modelling on small datasets, we introduced the Adaptive boosting (AdaBoost) [23], which improves the generalizability by combining weak base learners and bootstrap sampling with the AdaBoost algorithm.

Moreover, we introduced the RUSBoost and SMOTEBoost, which are ensemble learning methods based on AdaBoost with undersampling and oversampling, respectively, addressing both low generalizability and imbalance problems simultaneously.

In our comparative experiments, cost-sensitive SVM, SMOTEBoost, RUSBoost were applied to the original imbalanced dataset. SVM and AdaBoost were applied to the original imbalanced dataset and the balanced data balanced with ADASYN, respectively, to test the effectiveness of the data balancing method.

All classifier parameters were tuned with bootstrap fivefold cross-validation, and the decision tree was employed as the base learner for AdaBoost, RUSBoost and SMOTEBoost.

### Statistical analysis

Descriptive statistics are summarized as the mean $\pm$ standard deviation (mean $\pm$ std). Univariate analyses were performed on each feature of the training set using the *t*-test for 380 continuous radiomics features and the ${\upchi }^{2}$ test for two categorical clinical features. A *p* value < 0.05 indicated a significant difference.

Since our data is class imbalanced, the metrics used to evaluate the model's classification performance should be sensitive to class imbalance. The metrics we introduced in this study are the balanced accuracy (bACC), the area under the receiver operating characteristic (ROC) curve (AUC), the sensitivity (SENS), the specificity (SPEC), the positive predictive value (PPV) and negative predictive value (NPV). All methods were performed with MATLAB R2019b (MathWorks, Inc., Natick, MA, USA). The image processing toolbox and machine learning toolbox were applied in feature extraction and model building.

## Result

### Patient characteristics

A summary of the characteristics of the training set and test set is listed in Table 2. The imbalance ratio between the number of normal and NRM was close to 3:1. There is a significant difference (*p* value < 0.005) in both GA and GDM between NRM and normal controls, which is the statistical basis for using GA and GDM as clinical features. Moreover, there is a significant difference (*p* value < 0.0001) in birth weight between the two groups.

**Table 2:** Characteristics of the training set and test set

Characteristics	Training set (*n* = 167)			Test set (*n* = 43)		
	Normal	NRM	*p* value	Normal	NRM	*p* value
No. of images	127	40	–	32	11	
GA*	36.49 ± 0.85	34.37 ± 2.42	**< 0.0001**	36.78 ± 1.64	34.53 ± 2.37	**< 0.0001**
Birth weight (g)*	3096 ± 385	2978 ± 490	**< 0.0001**	3145 ± 423	3024 ± 540	**< 0.0001**
*GDM*			**< 0.005**			**0.06**
Yes	48 (37.80%)	26 (65.00%)	–	10 (31.25%)	7 (63.64%)	–
No	79 (62.20%)	14 (35.00%)	–	22 (68.75%)	4 (36.36%)	–
*Mode of delivery*			0.35			0.94
Spontaneous vaginal delivery	56 (44.09%)	21 (52.50%)	–	15 (46.88%)	5 (45.45%)	–
Caesarean delivery	71 (55.91%)	19 (47.50%)	–	17 (53.12%)	6 (54.55%)	–
*Sex of newborn*			0.87			0.43
Female	59 (46.46%)	18 (45.00%)	–	16 (50.00%)	7 (63.64%)	–
Male	68 (53.54%)	22 (55.00%)	–	16 (50.00%)	4 (36.36%)	–
*Apgar*			–			–
5 min ≤ 7	0(0.00%)	4 (10.00%)	–	0 (0.00%)	0 (0.00%)	–
5 min > 7	1 27 (100.00%)	36 (90.00%)	–	32 (100.00%)	11 (100.00%)	–

The  *p *value < 0.05 is shown in blod

The *t* test was performed for continuous variables and the χ^2^ test was performed for categorical variables

*GA* gestational age, *GDM* gestational diabetes mellitus

*Data are means ± standard deviations

### Univariate analysis and feature selection

Univariate analysis was performed on the training set. The results show that 32 of all 380 radiomics features were highly correlated with NRM (*p* value < 0.05).

**Fig. 4:**
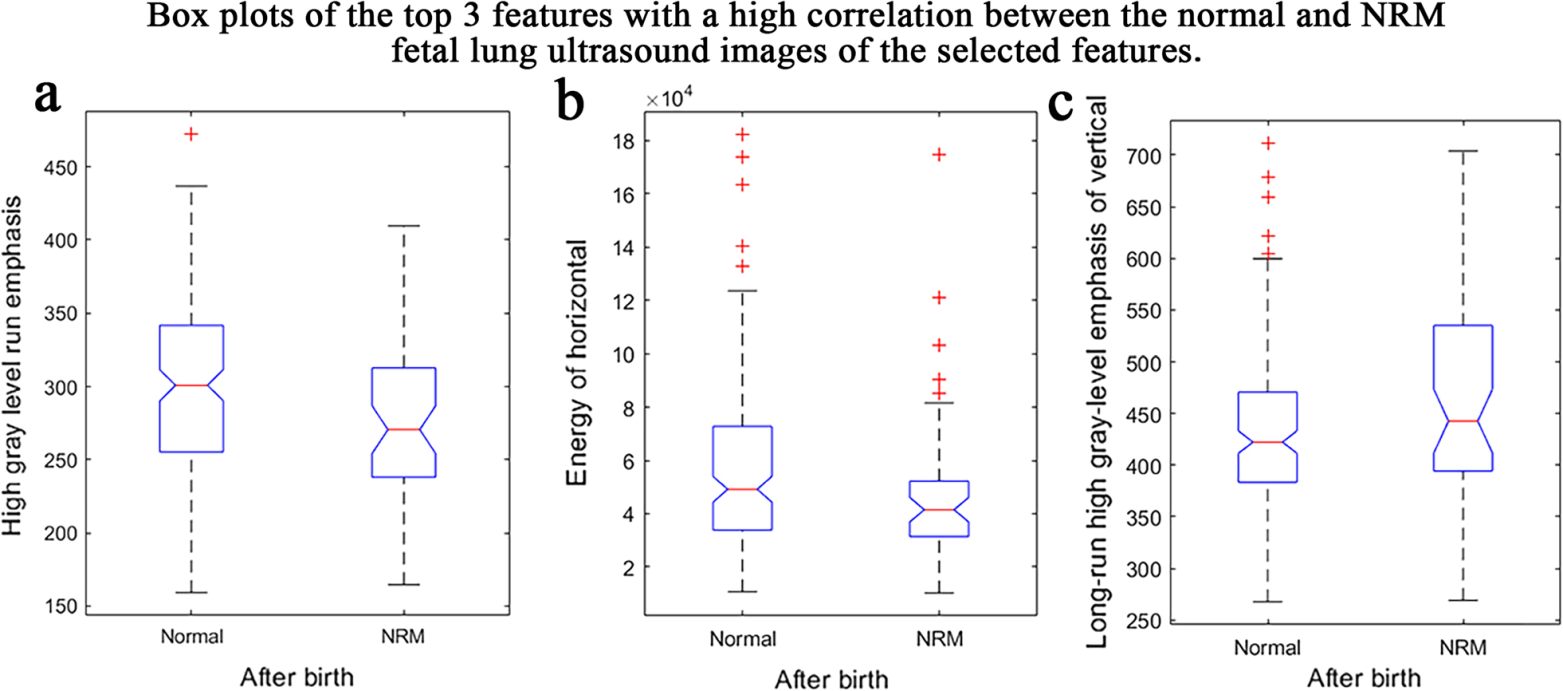
Box plots of the top 3 features of the 10 selected features. **a**–**c** are the box plots of the high grey-level run emphasis, energy of horizontal and long-run high grey-level emphasis of vertical features extracted from the ROIs of the normal and NRM samples. The normal fetal lung has higher mean values for the features of high grey-level run emphasis (298 ± 62.5) and energy of diagonal (1400 ± 724) than the NRM. For the long-run high grey-level emphasis of vertical feature, the mean value of the normal fetal lung is 432, which is smaller than that of the NRM of 462

The feature selection method was used to select the most useful features for modelling. The final 10 features with the highest feature's importance score were selected. The feature names and descriptive statistics of the 10 radiomics features selected are listed in Table 3. Figure 4 shows the box plots of the top 3 features with a high correlation between the normal and NRM fetal lung ultrasound images of the 10 selected features. Although there are significant differences in the means, the standard deviations overlap, making the classification task difficult and requires a more powerful multivariate classification method.

**Table 3:** Feature names and means of the features selected

Feature name	Mean ± std	
	Normal	NRM
Energy	0.543 ± 0.070	0.551 ± 0.063
Inverse difference moment normalized	0.999 ± 0.0004	0.998 ± 0.0005
High grey-level run emphasis	298 ± 62.5	279 ± 57.0
Run-length variance	(2.04 ± 0.996) × 10^−5^	(2.30 ± 0.844) × 10^−5^
Inverse difference moment normalized of approximation	0.801 ± 0.113	0.773 ± 0.114
Information measure of correlation 1 of approximation	0.989 ± 0.002	0.990 ± 0.002
Energy of horizontal	0.362 ± 0.036	0.374 ± 0.042
Sum entropy of vertical		(4.81 ± 2.87) × 10^4^
Long-run high grey-level emphasis of vertical	432 ± 78.4	462 ± 95.3
Energy of diagonal	(1.40 ± 0.724) × 10^3^	(1.20 ± 0.841) × 10^3^

Approximate, horizontal, vertical, and diagonal were decomposed from the image by wavelet transform (first-level decomposition)

### Model construction and evaluation

The classification performance of different modelling methods is illustrated in Table 4. The inputs to the model were 2 clinical features and 10 radiomics features, as shown in Table 3.

**Table 4:** The classification performance of different modelling methods

Method	Training set (mean ± std)						Test set					
	bACC	AUC	SENS	SPEC	PPV	NPV	bACC	AUC	SENS	SPEC	PPV	NPV
*Original imbalanced training set*												
SVM	0.66 ± 0.05	0.76 ± 0.07	*0.32* ± *0.11*	**0.99** ± **0.02**	**0.93** ± **0.07**	0.82 ± . 0.02	0.68	0.78	*0.36*	**1.00**	**1.00**	*0.82*
AdaBoost	0.76 ± 0.14	0.72 ± 0.16	0.68 ± 0.18	0.84 ± 0.09	09 ± . 0.09	**0.89** ± **0.05**	0.73	0.79	0.55	0.91	0.68	0.85
Cost-sensitive SVM	*0.66* ± *0.15*	0.73 ± 0.10	0.43 ± 0.21	0.89 ± 0.09	0.61 ± 0.17	0.84 ± 0.04	*0.65*	*0.75*	0.45	0.84	*0.49*	*0.82*
*Balanced training set augmented with ADASYN*												
SVM	0.71 ± 0.17	0.79 ± 0.10	0.67 ± 0.17	*0.74* ± *0.11*	0.45 ± 0.05	0.88 ± 0.04	0.76	0.85	0.73	0.78	0.53	0.89
AdaBoost	0.66 ± 0.14	0.71 ± 0.08	0.55 ± 0.15	0.76 ± 0.07	*0.42* ± *0.11*	0.85 ± 0.03	0.74	0.82	0.73	*0.75*	0.5	0.89
*Original imbalanced training set (combining data balance and ensemble learning)*												
SMOTEBoost	0.71 ± 0.11	*0.70* ± *0.09*	0.52 ± 0.14	0.89 ± 0.10	0.72 ± 0.18	0.85 ± 0.02	0.72	0.80	0.55	0.88	0.61	0.85
RUSBoost	**0.77** ± **0.10**	**0.83** ± **0.13**	**0.72** ± **0.15**	0.82 ± . 0.12	0.74 + 0.02	*0.82* ± *0.12*	**0.83**	**0.87**	**0.82**	0.84	0.64	**0.93**

The best results of each metric are shown in bold, and the worst results are shown in italics. Performance evaluation results obtained by bootstrap K-fold cross-validation in the training set

On the original imbalanced dataset, the SVM has a severe class bias, testing with a SPEC of 1.00 but a SENS of only 0.36. The cost-sensitive SVM model obtains a small increase in SENS of 0.36–0.45 but is accompanied by a large decrease in SPEC of 1.00–0.84. The AdaBoost shows a better performance than the cost-sensitive SVM, while SPEC decreased by only 0.09.

Training the SVM and AdaBoost models on the balanced dataset resulted in a substantial increase in SENS compared to the results from the original imbalanced dataset, both reaching 0.73, but correspondingly, a substantial decrease in SPEC, from 1.00 to 0.78 and from 0.91 to 0.75, respectively.

The SMOTEBoost's SENS is equal to that of the AdaBoost trained on the original imbalanced dataset, but its SPEC is only 0.88, lower than AdaBoost's 0.91. RUSBoost shows better classification performance than other methods, with a SENS of 0.72, a SPEC of 0.82, a bACC of 0.77, and an AUC of 0.83 by bootstrap validation in the training set. Moreover, the model has excellent classification performance with a SENS of 0.82, a SPEC of 0.84, a bACC of 0.83, and an AUC of 0.87, in the test set.

### The effect of our feature set

The verification result of feature set effectiveness is illustrated in Table 5. In the test set, the model built with the feature of GA alone has a high SPEC of 0.97 and a low SENS of 0.45. For the combination of GA and GDM, there is an increase in SENS from 0.45 to 0.69, but SPEC decreases by 0.34. The best classification performance can be achieved with our designed feature set, including radiomics features, GA, and GDM.

**Table 5:** The classification performance of RUSBoost with different features on the original imbalanced few-shot dataset

Feature	Training set (mean ± std)						Test set					
	bACC	AUC	SENS	SPEC	PPV	NPV	bACC	AUC	SENS	SPEC	PPV	NPV
GA	0.72 ± 0.11	*0.80* ± *0.10*	*0.58* ± *0.21*	**0.88** ± **0.06**	0.60 ± 0.11	0.87 ± 0.08	0.71	**0.97**	*0.45*	**0.97**	**0.83**	*0.84*
GA & GDM	*0.71* ± *0.15*	**0.83** ± **0.10**	0.72 ± 0.24	*0.69* ± *0.15*	*0.42* ± *0.14*	0.89 ± 0.15	*0.66*	*0.83*	0.64	*0.68*	*0.41*	0.85
*Radiomics features extracted from the irregular ROI*												
GA, GDM & Radiomics	**0.77** ± **0.08**	0.83 ± 0.13	**0.72** ± **0.05**	0.82 ± 0.12	**0.74 + 0.02**	*0.82* ± *0.12*	0.83	0.87	0.82	0.84	0.64	0.93
*Radiomics features extracted from the square ROI*												
GA, GDM & Radiomics	0.76 ± 0.08	0.81 ± 0.09	0.70 ± 0.07	0.82 ± 0.15	0.55 ± 0.04	**0.90** ± **0.09**	**0.87**	0.89	**0.91**	0.82	0.63	**0.96**

The best results of each metric are shown in bold, and the worst results are shown in italics. Performance evaluation results obtained by bootstrap K-fold cross-validation in the training set.

**Fig. 5:**
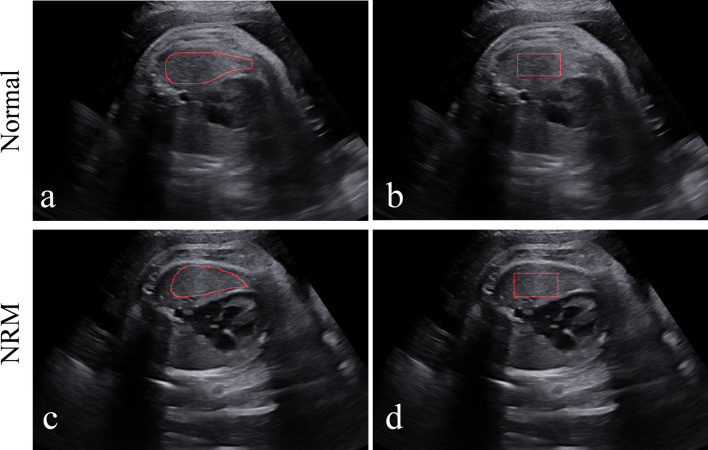
Examples of the lung region delineations in the lung ultrasound images of a normal fetus and a fetus with NRM. **a** and **b** are the irregular and square ROI selection in the ultrasound image of a normal fetus. **c** and **d** are the irregular and square ROI selection in the ultrasound image of a fetus with NRM

Since most areas inside the fetal lung are homogeneous, the greyscale histogram features and texture features have the stability for small changes of the location or shape of the ROI in the homogeneous region. As a validation measure of the stability of the feature set, each image is additionally delineated with a square ROI in addition to the irregular ROI. The square ROI was outlined within the fetal lung region, as shown in Fig. 5. As illustrated in Table 5, the irregular ROI and square ROI achieved similar performance outcomes. There is only a difference of 0.04 in bACC, 0.02 in AUC, 0.09 in SENS, 0.02 in SPEC, 0.01 in PPV, and 0.03 in NPV on the test set. These results demonstrate our texture feature-based model has the stability for the shape and location of the ROI.

**Fig. 6:**
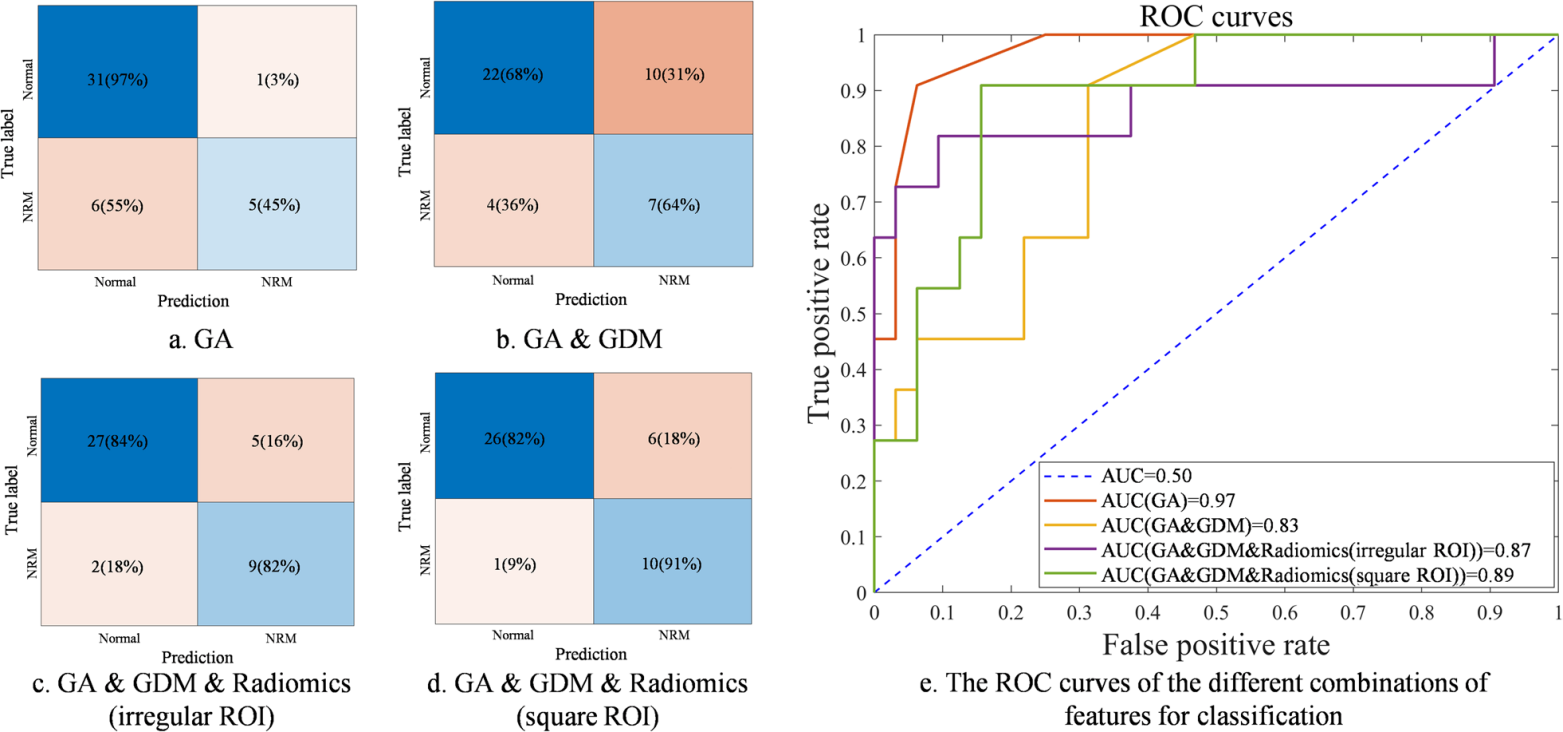
The confusion matrix and ROC curves tested in the test set with different combinations of features. **a** and **b** are confusion matrices of the model using only clinical data. **c** is confusion matrices of the model using clinical data combined with delineated ROI. **d** is confusion matrices of the model using clinical data combined with square ROIs. **e** shows ROC curves and AUC values for different combinations of features

As shown in Fig. 6a and b, clinical models have severe class bias, leading to low sensitivity. In the model using GA, only 45% of the NRM samples were correctly diagnosed. In the model using GA and GDM, only 64% of the NRM samples were correctly diagnosed. Model using both clinical data and radiomics features achieves the best diagnostic performance, as illustrated in Fig. 6c and d. There are 82% and 91% of NRM samples correctly diagnosed, respectively, while less than 20% of Normal samples were misdiagnosed as NRM. Furthermore, in Fig. 6e, tests using different ROIs achieved similar classification performance and ROC curves. It is worth to be noted that the classifier using GA only or GA and GDM is biased towards the normal class, while its AUC is higher due to the imbalance of the dataset, which is the limitation of AUC in the classification performance evaluation of imbalanced datasets.

## Discussion

Prenatal prediction and therapy for NRM are an effective way to improve the life quality of NRM newborns. There is a consensus to study non-invasive methods to predict NRM using fetal lung ultrasound images. However, there is no unified feature set for the prenatal prediction of NRM, and the dataset collected in medical practice is often imbalanced and few-shot. To tackle these challenges, our study focuses on the design of feature sets with a strong representation of fetal lung ultrasound images and effective classification modelling methods.

### The feature set for predicting NRM

Considering that the fetal lung in the ultrasound image is homogeneous, we designed radiomics features based on the image greyscale and texture, which can avoid the influence of the ROI's size and location on feature extraction. For each fetus, 380 radiomics features were extracted from the fetal lung region of ultrasound images, and 10 of them were selected for modelling. The energy of horizontal, which characterizes the brightness in the horizontal direction of the wavelet transform, has a mean value of 1400 in normal fetal lungs, which is higher than 1200 in NRM fetal lungs. The high grey-level run emphasis of the normal fetal lung has a higher mean value of 298 than the NRM fetal lungs of 279, which means that the fetal lung region is more homogeneous in normal fetal lungs than NRM fetal lungs. For the long-run high grey-level emphasis of vertical feature, the mean value of the normal fetal lungs is 432, which is smaller than that of the NRM fetal lung of 462, which suggests that the fetal lung region is more delicate in normal fetal lungs than NRM fetal lungs. It can be concluded that the lung region of normal fetuses has a more delicate and homogeneous texture on the ultrasound image and is brighter than that of NRM fetuses. The features we selected were also stable. The radiomics features extracted from the irregular ROI and the square ROI achieved similar performance outcomes with the same modelling method (the difference was less than 0.09 for each measure), as shown in Table 5.

In addition to radiomics features, GA and GDM, two clinical features identified to be strongly correlated with NRM, were also added to the feature set. Newborns with a low GA have a significantly increased risk of NRM due to immature lungs, and GDM in pregnant women leads to delayed lung development in the fetus, increasing the risk of NRM. As shown in Table 5, with the addition of radiomics features, the SPEC and SENS were both significantly improved. In conclusion, the feature set designed in this study that includes radiomics features, GA, and GDM is more effective for NRM prediction and is not affected by the size or location of the ROI.

### Model development

Imbalance and few-shot are inevitable in medical datasets, which pose many challenges for modelling. As shown in Table 4, there is a large class bias and poor classification performance on small imbalanced datasets using the conventional SVM. The methods of data augmentation, cost-sensitive learning, and ensemble learning are commonly used on imbalanced few-shot datasets. Here, these methods were performed and analysed to find the most effective modelling method.

The cost-sensitive SVM and AdaBoost show an improvement of 0.21 and 0.36 in SENS compared with the SVM in Table 4, but there is a decrease of 0.10 and 0.15 in SPEC in the training set. As for the cost-sensitive SVM, since there are few NRM samples, a higher cost is needed, which makes the compression of boundaries more severe, and the classifier tends to sacrifice multiple normal samples to ensure that one NRM sample is correct with a sharp decline in the generalization performance. The AdaBoost has a better performance than cost-sensitive SVM, with a SENS of 0.68 and a SPEC of 0.84. The ensemble learning method's lower overfitting allows it to exhibit a better generalization performance than the individual learner SVM or the cost-sensitive SVM.

**Fig. 7:**
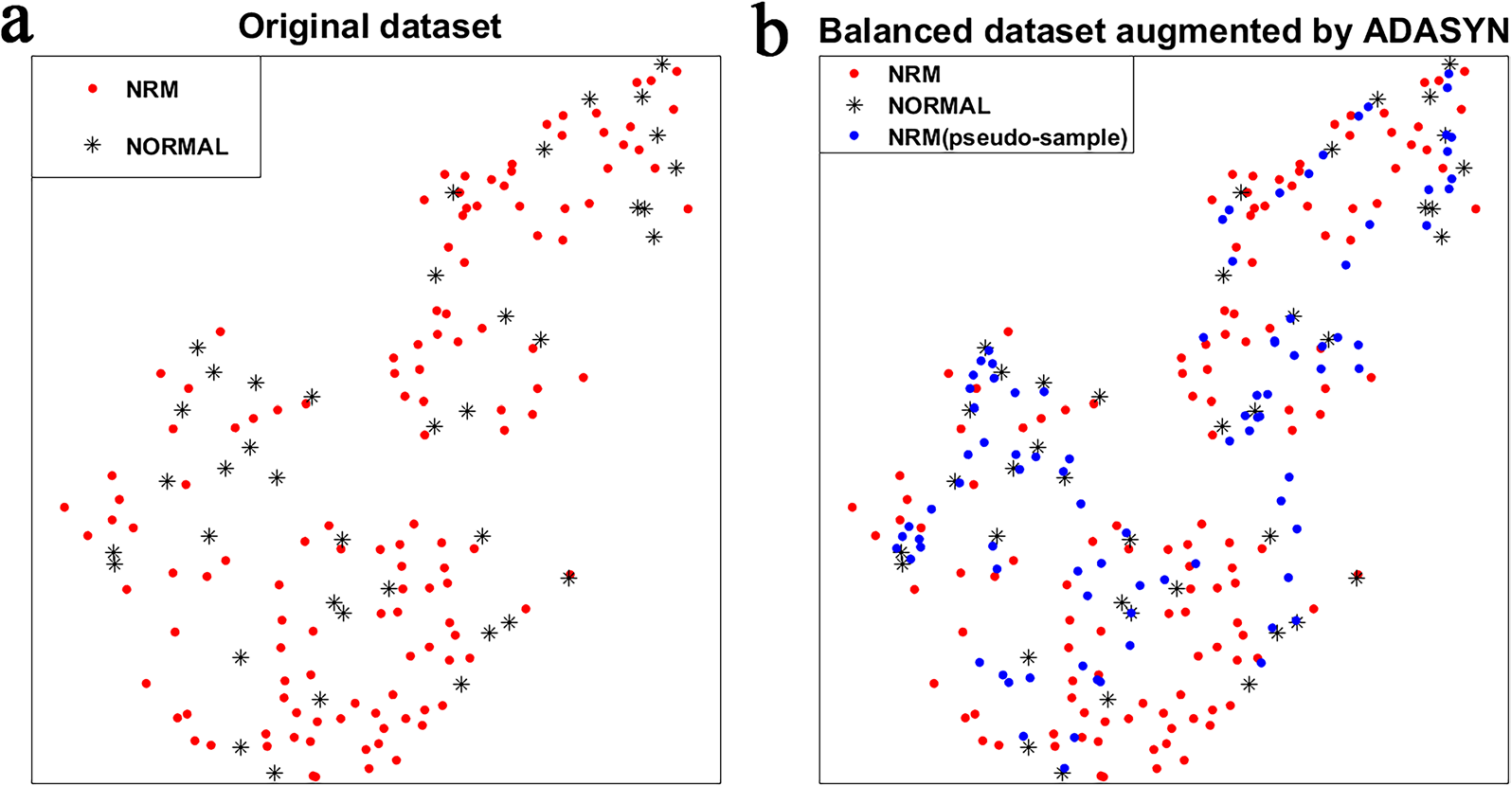
The distribution of the samples. **a** The sample distribution of the original dataset with terrible class aliasing. **b** The sample distribution of the balanced dataset augmented by ADASYN

Training on the balanced training set augmented with ADASYN, the SVM and AdaBoost does not show a significant improvement compared to training on the original imbalanced dataset, with an increase of 0.35 and 0.23 in SENS and a decrease of 0.25 and 0.26 in SPEC. For better illustration, we used t-SNE [24] to visualize the sample distribution of the original dataset and the balanced dataset augmented by ADASYN. As shown in Fig. 7, there is aliasing between normal and NRM samples, making it difficult to classify. By generating pseudo-samples around the minority class, ADASYN leads the classifier to draw more attention to the NRM samples. However, it also exacerbates aliasing and results in poor classification performance. The generated pseudo-samples also tend to introduce plenty of noise, especially when the aliasing of samples is terrible. The data augmentation method is not appropriate in our application.

The SENS of SMOTEBoost is still low because aliasing in the dataset makes SMOTE introducing considerable noise. RUSBoost shows better classification performance than other methods. It reaches a SENS of 0.72, a SPEC of 0.82, a bACC of 0.77, and an AUC of 0.83 in the training set and a SENS of 0.82, a SPEC of 0.84, a bACC of 0.83, and an AUC of 0.87 in the test set. RUSBoost can reduce overfitting and improve the classification model's generalization ability by combining weak base learners and bootstrap sampling with the AdaBoost algorithm. The input dataset of each learner is obtained by bootstrap undersampling, which enriches the sample distribution that the base learners have learned and reduce the effects of imbalance. The drawback of massive sample loss of undersampling in a small dataset is compensated by ensemble learning, while random undersampling ensures that the samples are real and avoids the noise that caused by data augmentation.

### NRM prediction model

In this study, the non-invasive approach we proposed based on the Asian population utilizes a much smaller data set to establish similar prediction performance to previously reported methods. It makes it possible to safely and widely perform the NRM prenatal screening and intervention, which has an excellent prediction performance with a bACC of 0.83, an AUC of 0.87, a SENS of 0.82, a SPEC of 0.84, a PPV of 0.64, and an NPV of 0.93. A comparison of our method with some of the existing reported methods is illustrated in Table 6, which shows that our diagnostic performance approximates to that of invasive amniocentesis tests. Compared our study to Bonet' work [5], in which only fetal lung ultrasound images were used for NRM prediction, our method utilizes less than 1/2 of the training set size. There is 0.02 higher in bACC, 0.07 higher in SENS, 0.02 higher in NPV, the same PPV and only 0.04 lower in SPEC, with square ROI. Compared to quantusFLM [5] reported in a multicenter study, our study uses less than 1/4 of the training set size and is 0.05 higher in bACC, 0.17 higher in SENS, 0.12 higher in PPV, the same NPV and only 0.07 lower in SPEC, with square ROI. Our model based on the Asian population utilizes a much smaller data set to establish better prediction performance to previously reported methods.

**Table 6:** Comparison of our method with previously reported methods

Method	Size of training set	Test set				
		bACC	SENS	SPEC	PPV	NPV
TDxII [5]	-	*0.82*	0.86	*0.78*	*0.29*	**0.99**
Bonet [25]	N = 390 (NRM: -)	0.85	0.84	0.86	0.63	0.94
quantusFLM [5]	N = 730 (NRM: 13.8%)	*0.82*	*0.74*	**0.89**	0.51	0.96
Our method (irregular ROI)	N = 167 (NRM: 24.0%)	0.83	0.82	0.84	0.64	*0.93*
Our method (square ROI)	N = 167 (NRM: 24.0%)	**0.87**	**0.91**	0.82	0.63	*0.96*

The best results of each metric are shown in bold, and the worst results are shown in italics

TDxII, surfactant/albumin ratio, the best index in the report of amniocentesis results

In Bonet's work, SENS, SPEC, PPV, and NPV were calculated at different gestational week groups, and the table shows the mean values

Our model was built and tested in female and male fetuses with GAs ranging from 28.0 to 38.6 weeks. The experimental results show that our model has effective predictive performance in this scope. Moreover, our method has a degree of stability for the ROI's location and shape, allowing the model to be widely used.

### Strengths and limitation

Our study has three strengths. First, to the best of our knowledge, this is the first study to incorporate GDM, GA, and radiomics features for NRM prenatal prediction. The diagnostic efficacy of the model we developed based on fetal lung ultrasound images in this study reached which are similar to those of many previous reports of amniocentesis [26–28]. Second, we developed a practical modelling approach to address the problems of imbalance and few-shot. RUSBoost shows excellent performance and generalization capabilities compared with the other methods used for comparison in this study. Third, we used radiomics features based on the image greyscale and texture for the prenatal prediction of NRM, whose performance is efficient and robust, without the influences of ROI selection results.

As a retrospective study, this study has some limitations that should be acknowledged. Clinical outcome of the fetuses depends on several clinical factors. In addition to GA and GDM, more clinical information could be studied for its correlation with fetal lung development and used for NRM prediction. A comparative study on the right and left lungs to verify the generalizability of the method between the right and left lungs is also needed. Furthermore, for applying the proposed method to a clinical application, a robust validation technique is required to demonstrate the stability of our model on the multicenter dataset from different machines and different operators. The applicable fetal population (different gestational week groups or sexes) is also needed to be investigated in our upcoming multicenter experiment.

In order to answer these questions and overcome these limitations, a multicenter study is underway. Additional fetal ultrasound images from multicenter will be included in our study for robust validation.

## Conclusion

In conclusion, our results show that the radiomics features of the fetal lung can be used as an efficient and robust biomarker for NRM prediction. The diagnostic efficacy of the model based on fetal lung ultrasound images, which incorporates routinely available clinical characteristics GA and GDM and radiomics features, achieves a better clinical outcome, which might afford a non-invasive tool that is easy to implement in NRM prediction.

## Data Availability

The datasets generated and analysed during the current study are not publicly available due to the data being also a part of an ongoing study but are available from the corresponding author on reasonable request.

## Abbreviations

NRM: Neonatal respiratory morbidity
RDS: Respiratory distress syndrome
TTN: Transient tachypnea of the newborn
HRV: Heart rate variability
GA: Gestational age
quantusFLM: Quantitative texture analysis of fetal lungs
GDM: Gestational diabetes mellitus
ROI: Region of interest
RUSBoost: Random under-sampling with AdaBoost
SMOTE: Synthetic minority oversampling technique
SMOTEBoost: SMOTE with AdaBoost
IBSI: Imaging biomarker standardization initiative
db5: Daubechies wavelets 5
ADASYN: Adaptive synthetic
SVM: Support vector machine
AdaBoost: Adaptive boosting
bACC: Balanced accuracy
ROC: Receiver operating characteristic
AUC: Area under the ROC curve
SENS: Sensitivity
SPEC: Specificity
PPV: Positive predictive value
NPV: Negative predictive value
